# Application of the Improved Rapidly Exploring Random Tree Algorithm to an Insect-like Mobile Robot in a Narrow Environment

**DOI:** 10.3390/biomimetics8040374

**Published:** 2023-08-17

**Authors:** Lina Wang, Xin Yang, Zeling Chen, Binrui Wang

**Affiliations:** 1 College of Mechanical and Electrical Engineering, China Jiliang University, Hangzhou 310018, China; 2 Key Laboratory of Intelligent Manufacturing Quality Big Data Tracing and Analysis of Zhejiang Province, China Jiliang University, Hangzhou 310018, China

**Keywords:** mobile robot, path planning, RRT algorithm, target bias sampling, adaptive step size

## Abstract

When intelligent mobile robots perform global path planning in complex and narrow environments, several issues often arise, including low search efficiency, node redundancy, non-smooth paths, and high costs. This paper proposes an improved path planning algorithm based on the rapidly exploring random tree (RRT) approach. Firstly, the target bias sampling method is employed to screen and eliminate redundant sampling points. Secondly, the adaptive step size strategy is introduced to address the limitations of the traditional RRT algorithm. The mobile robot is then modeled and analyzed to ensure that the path adheres to angle and collision constraints during movement. Finally, the initial path is pruned, and the path is smoothed using a cubic B-spline curve, resulting in a smoother path with reduced costs. The evaluation metrics employed include search time, path length, and the number of sampling nodes. To evaluate the effectiveness of the proposed algorithm, simulations of the RRT algorithm, RRT-connect algorithm, RRT* algorithm, and the improved RRT algorithm are conducted in various environments. The results demonstrate that the improved RRT algorithm reduces the generated path length by 25.32% compared to the RRT algorithm, 26.42% compared to the RRT-connect algorithm, and 4.99% compared to the RRT* algorithm. Moreover, the improved RRT algorithm significantly improves the demand for reducing path costs. The planning time of the improved RRT algorithm is reduced by 64.96% compared to that of the RRT algorithm, 40.83% compared to that of the RRT-connect algorithm, and 27.34% compared to that of the RRT* algorithm, leading to improved speed. These findings indicate that the proposed method exhibits a notable improvement in the three crucial evaluation metrics: sampling time, number of nodes, and path length. Additionally, the algorithm performed well after undergoing physical verification with an insect-like mobile robot in a real environment featuring narrow elevator entrances.

## 1. Introduction

In an era characterized by the increasingly mainstream development of artificial intelligence, mobile robots have gained popularity in various fields, such as autonomous driving and intelligent factories. As a significant focus of robot learning, mobile robot path planning has become a topic that is widely discussed by experts and scholars. Path planning for mobile robots can be categorized into traditional path planning algorithms, intelligent path planning algorithms, and sample-based planning algorithms [1]. Traditional path planning algorithms include methods such as artificial potential fields, bug algorithms, vector field histograms, and raster methods. Commonly used algorithms in map rasterization and robot path planning are the A* algorithm [2] and the Dijkstra algorithm [3]. Intelligent path planning algorithms encompass techniques such as the ant colony algorithm [4], genetic algorithm [5], neural network algorithm [6], and others. In the realm of intelligent planning algorithms, path planning can be further divided into global path planning and local path planning. Global path planning aims to devise a feasible path from the starting point to the target point, without considering dynamic obstacles, when the map environment information is known. On the other hand, the local path planning algorithm deals with temporary obstacles that may appear along the global path, impeding the normal movement of the robot. In such cases, the algorithm plans a path for local obstacle avoidance. Global path planning algorithms must be able to find collision-free paths across the entire environment space, while also meeting conditions such as minimum path cost, minimum planning time, and minimum cost. However, both traditional path planning algorithms and intelligent path planning algorithms depend on the representation and handling of obstacles and require structural treatment. The most widely used sample-based search algorithm is the rapidly exploring random tree (RRT) [7], which was proposed by Lavalle [8] in 1998. The rapidly exploring random tree algorithm, based on node sampling, offers the advantages of simple obstacle modeling, making it suitable for path planning of robotic arms in complete systems, wheeled mobile robots, UAVs in non-holonomic systems, and others. Its greatest advantage lies in its ability to operate without the need for space preprocessing, ensuring probabilistic completeness. However, the rapidly exploring random tree algorithm still exhibits several shortcomings in path planning [9]: blindness, randomness, and non-directivity in searching; low node utilization, resulting in redundant nodes throughout the search process; slow convergence speed and low search efficiency; and paths that are feasible but not optimal. In response to these issues, many experts and scholars have devoted themselves to improving the deficiencies of the RRT algorithm. To address the problem of slow convergence, Lavalle and Kuffner proposed the RRT-connect algorithm [10], which connects two random trees through a greedy search to reduce the number of sampling nodes [11] and ultimately achieve a higher convergence speed. In light of the issue that the path obtained by the RRT algorithm is feasible but not optimal, Karama and Frazzoli proposed the RRT* algorithm [12], which incorporates path cost information and a rewiring operation. The resulting path exhibits progressive optimality and can converge to the optimal path given enough time, but it comes at the expense of increased convergence time and reduced speed. Furthermore, numerous RRT series algorithms have been developed as part of ongoing research, including the KDT-RRT algorithm [13], the PG-RRT algorithm [14], and the DGABI-RRT algorithm [15]. To enhance trajectory quality, Chang-bae Moon et al. [13] proposed the KDT-RRT algorithm, also known as the dynamic double-tree fast exploration random tree algorithm. This algorithm modifies the reconnection tree of the spanning tree structure without the need for retransmission found in the traditional RRT structure. By utilizing different tree structures, flexible node expansion is achieved, resulting in improved path quality. To address the slow convergence problem, Sharma Paras et al. [14] proposed the PG-RRT algorithm, an intelligent probabilistic Gaussian mixture model-driven algorithm. This approach generates nodes in regions with the highest probabilities in the model, leading to faster convergence. Li et al. [15] proposed the DGABI-RRT algorithm, aiming to obtain a better path with a reduced path cost and a smooth trajectory. This algorithm adopts an improved sampling point strategy and a gravity adaptive step size method to introduce randomness, enabling the mobile robot to exhibit target orientation and obstacle avoidance in its movement. The path optimization strategy further enhances the smoothness of the path. Building upon the advancements in sampling point improvement and adaptive step size strategies, this paper presents a more direct and efficient method for trajectory optimization. It calculates the maximum proportion of effective points needed to eliminate redundant points, adjusts the exploration extension length adaptively based on the environment, and does not rely solely on its quality to determine the extension distance. Additionally, many RRT series algorithms are combined with other techniques to create new algorithms aimed at achieving optimization. Feng Laichun et al. [16], recognizing challenges such as low sampling efficiency and slow speed, combined the A* algorithm with the RRT algorithm to construct a guide domain using the shortest path generated by the A* algorithm in a raster graph. This integration improves the sampling efficiency of the RRT algorithm. Another approach proposed by Zang Qiang et al. [17] involves enhancing the relative distance potential field method by fusing it with the adaptive artificial potential field method and the RRT* algorithm. This fusion significantly enhances path efficiency and flexibility for obstacle avoidance. Drawing from existing research results, this paper proposes an improved RRT algorithm for global path planning of mobile robots, addressing issues such as sampling point redundancy, low search efficiency, high path cost, and uneven paths. The algorithm initially employs a greedy target bias sampling method to mitigate the excessive randomness of sampling points in the RRT algorithm’s sampling process. Additionally, the algorithm introduces an adaptive step size strategy while considering non-integrity constraints such as vehicle collision and corner cases. In contrast to using a gravity adaptive step size or expanding the step angle with the target, this paper adopts objective bias sampling to enhance the quality of effective sampling points. Furthermore, redundancy point elimination optimization is achieved by ensuring the maximum proportion of effective points is reached. Finally, the improved path is pruned and smoothed to obtain a safe and smooth trajectory that can be easily tracked by the robot.

## 2. RRT Algorithm Based on Adaptive Step Size Strategy

**Figure 1:**
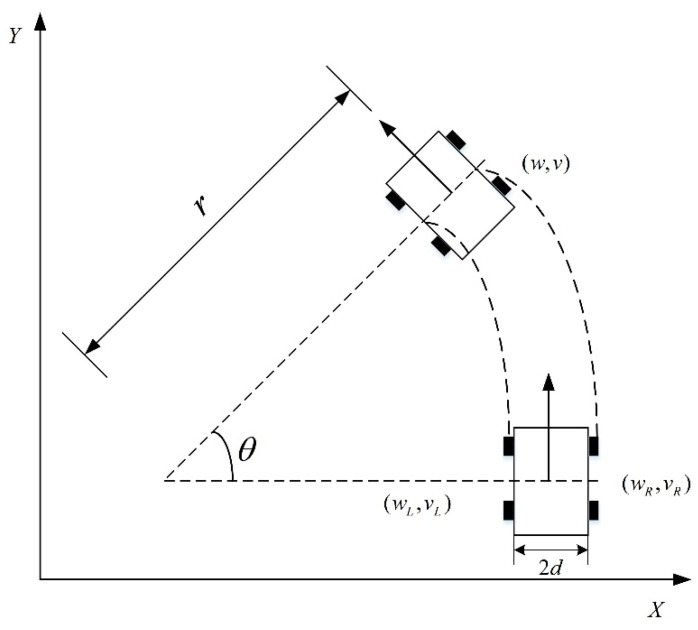
Differential modeling of mobile robot.

The RRT algorithm can only find a path that connects the starting point and the ending point. However, the planned path is convoluted and does not adhere to the kinematic constraints of the mobile robot’s vehicle body [18], making it impossible for the vehicle to follow the intended global path. Despite the mobile robot movement platform utilizing a four-wheel movement chassis, its structure consists of fixed wheels added to a two-wheel differential chassis. To navigate uneven terrain, chains are installed on both sides of the vehicle body, enabling the movement of four wheels driven by two motors. The robot is analyzed and modeled using a kinematic model of the two-wheel differential chassis. The insect-like mobile robot kinematic model is illustrated in Figure 1.

### 2.1. RRT Algorithm

During the process of implementing path planning movement, mobile robots are required to make fundamental judgments and selections based on the environment map [19]. To identify and process environmental information effectively, various environmental map models are utilized. Commonly used environmental map models include the raster map model, feature map [20] model, and topological map model and three map models have been shown in Table 1.

This paper utilizes the map of the mobile robot’s environment as the foundation for algorithm research to represent the randomness of map information, obstacle information, and path planning. In global path planning, the starting point and target point are determined, and an environment with a randomized map size, static obstacle rules, and irregularities is constructed to verify the universality and scientific nature of the algorithm. A topological map is adopted, and the point-line structure is used to represent the map, providing an intuitive visualization that fully utilizes the advantages of the path planning algorithm based on sampling points.

**Figure 2:**
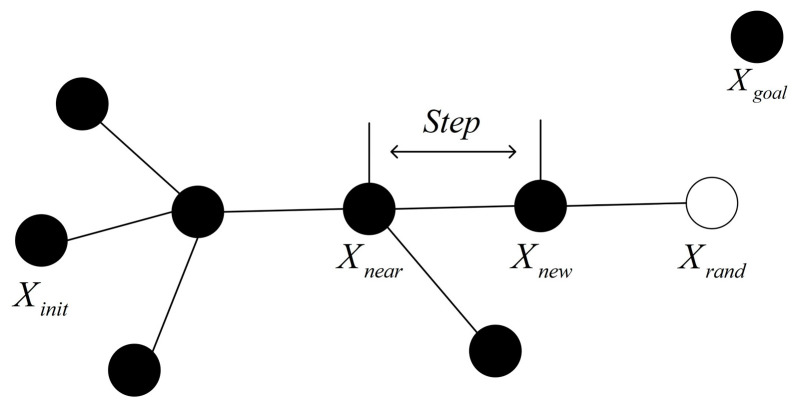
Schematic picture of RRT algorithm.

The traditional rapidly exploring random tree (RRT) algorithm [21] constructs a search random tree based on the initial pose and target pose obtained from the scene map. A schematic diagram of the RRT algorithm is shown in Figure 2. The starting point serves as the root node of the random tree. Through random sampling, the extension of the random tree is guided towards the free area for random scattering until the target point is reached. This process forms a random tree with path extensions, ultimately generating a globally planned path from the starting point to the target point.

The basic steps are as follows:

1. The mobile robot constructs a random tree at the starting point $X_{init}$ of the two-dimensional state space as the root node;
2. A random sampling point $X_{rand}$ is generated in the free search space and used to guide the expansion of the random tree;
3. After traversing the nodes that have been generated in the whole random tree, the tree node that is closest to the random point $X_{rand}$ is found and selected and defined as $X_{near}$;
4. From the node $X_{near}$ along the extension direction of the node $X_{rand}$ as the extension direction, the appropriate step size is expanded and an appropriate step size is set as the branch length to generate a new node $X_{new}$ as the new tree node;
5. If an obstacle is encountered in the expansion process, the expansion is canceled and sampling is performed again. The path repeats the above iterative process until the target node exceeds the specified number of iterations and finally forms a fast-expanding random tree path, ending the search. Presented below is Algorithm 1, which provides the fundamental pseudocode of the RRT algorithm.

**Algorithm 1:** RRT algorithm**Input:** $M,x_{init},x_{goal}$ **Result:** A path Γ from $x_{init}$ to $x_{goal}$
  1:τ.init();  2:For i=1 to n, do  3:$x_{rand}\leftarrow Sample(M)$;  4:$x_{near}\leftarrow Near(x_{rand},\tau )$;  5:$x_{new}\leftarrow Steer(x_{rand},x_{near},StepSize)$;  6:$E_{i}\leftarrow Edge(x_{new},x_{near})$;  7:If $CollisionFree(M,E_{i})$, then  8:$\tau .addNote(x_{new})$;  9:$\tau .addEdge(E_{i})$;10:If $x_{new}=x_{goal}$, then11:Success();12:End13:End

The traditional rapidly exploring random tree (RRT) algorithm constructs a search random tree based on the initial pose and target pose obtained from the scene map. A schematic diagram of the RRT algorithm is shown in Figure 2. The starting point serves as the root node of the random tree.

### 2.2. Target Bias Sampling Method

The traditional RRT algorithm is a randomized algorithm, resulting in a large number of redundant nodes during the sampling process. To address the issue of excessive sampling points and reduce the algorithm’s search process blindness, the growth of the random tree is controlled by random sampling across the entire map. However, the randomness of the sampling leads to the generation of numerous sampling nodes, which in turn slows down the convergence of the algorithm. Thus, it is crucial that sampling points that steer the random tree toward the target point are selected, optimizing the search speed. In the expansion process of RRT algorithm nodes, $X_{new}$ is obtained from the process of $X_{near}$ extending to $X_{rand}$. And it can be expressed as Equation (1):

(1) $$X_{new}=X_{near}+step\cdot \frac{X_{rand}-X_{near}}{d(X_{rand},X_{near})}$$

In Equation (1), step is the extended step size, and $d(X_{rand},X_{near})$ is the Euclidean distance from the node $X_{rand}$ to the state node $X_{near}$.

**Figure 3:**
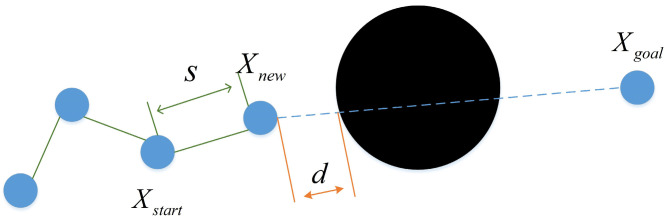
Target bias sampling.

It can be seen from Equation (1) that every node expansion generation process is accompanied by a large number of samples. Therefore, to improve the state sampling points, this paper proposes a greedy target bias sampling method [22], which reduces the blindness of selecting $X_{rand}$, as shown in Figure 3. The algorithm generates new nodes in each sampling process and detects the distance d between the new node and the nearest obstacle in the direction of the target point in each iteration. If the distance d is greater than or equal to the growth step size, the target point is regarded as the random point of the next sampling. If it is smaller than the growth step size, random sampling will be conducted in the map and random trees will grow randomly to bypass obstacles in the fastest way [23]. As shown in Figure 3, the distance is smaller than the growth step, so random sampling is needed to generate sampling point $X_{rand}$. The improved formula of target bias sampling is shown in Equation (2):

(2) $$X_{rand}=\left\{ \begin{array}{l} X_{goal}\;(d\geq step)\\ X_{rand}\;(d<step) \end{array}\right.$$

### 2.3. Adaptive Step Size Strategy

A variant step size is a good way for the RRT algorithm to improve the fixed step size. For example, in 2019, Cao [24] et al. improved the sampling point and extended step size in their paper. The measure of variable step size in this paper is the fitness function C=m/s+nD, where s represents the path length and D represents the distance between the manipulator and obstacles. A greater fitness function value is closer to the optimal solution, so by changing the values of the weighted factors m and n, paths of different importance can be obtained. However, variable step size selection is mostly performed through the superposition method or through the allocation of weight proportion, which is not convenient and has low efficiency. In this paper, we use the rising characteristic of the index to carry out proportional multiplication, and the effect is better than that achieved with other algorithms.

**Figure 4:**
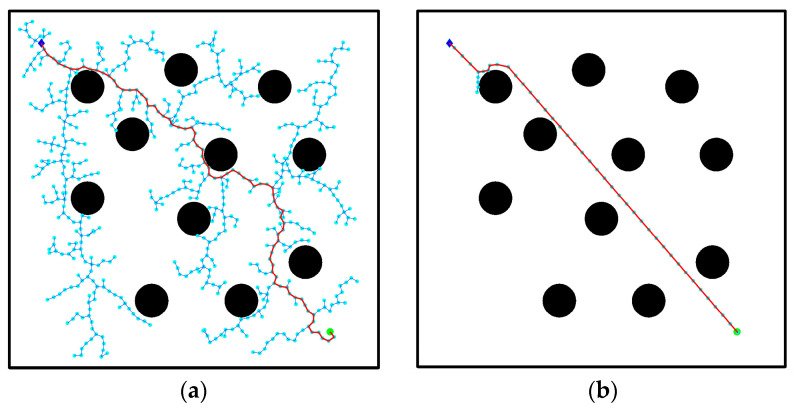
Improved algorithm comparison: (**a**) RRT algorithm obstacle avoidance effect; (**b**) adaptive step size algorithm obstacle avoidance effect.

This fixed sampling method poses a challenge when new nodes encounter more obstacles during their generation and expansion, as it prevents them from traversing the environment completely. Conversely, if there are fewer obstacles, the fixed sampling method leads to unnecessary node sampling, impeding quick traversal of the map. To address this issue, the step size of the random tree can be adjusted to accommodate various obstacle environments. Specifically, an adaptive step size can be generated based on the number and complexity of obstacles in specific circumstances [25]. This paper employs an adaptive step size strategy that utilizes information collected by the algorithm to dynamically adjust the step size in real time, enabling efficient traversal through complex narrow areas. The formula for selecting the adaptive step size is represented by Equation (3):

(3) $$step=s\times e^{k(\frac{N_{1}}{N})}$$

where s is the initial step size, k is the control coefficient, $N_{1}$ is the number of effective sampling points, and N is the total number of sampling points. To account for the complexity of the environment, a sampling forgetting counting scheme has been implemented. This scheme involves re-counting every sample three times. When the number of effective sampling points $N_{1}$ is greater, that is, the obstacle environment is simpler and there are fewer nearby obstacles, the generated step size will be larger. Otherwise, the step size is smaller. The number of nodes and the path planning time are shown in Table 2. Figure 4 presents a simple barrier-free environment with a small number of circular obstacles, and the path comparison generated by the search for fixed step size and improved dynamic step size is also shown.

As can be observed in Figure 4, the path generated in Figure 4b was significantly improved after incorporating adaptive step size and adjusting the step length of the expansion path. This enhancement reduces redundant choices of path nodes compared to the path depicted in Figure 4a. While the trajectory turning point may not be entirely smooth, it becomes possible to plan a path with a low cost and fewer interruptions while avoiding obstacles. The simulation results demonstrate that the quality of path planning can be greatly enhanced through effective node centralized processing. This not only reduces the convergence time but also enables the generation of a feasible trajectory by navigating real-time obstacle environments.

### 2.4. Path Pruning Optimization and Smoothing Treatment

The improved RRT algorithm can explore the obstacle avoidance path of the mobile robot, but a collision-free path is still limited to multiple turning points. To reduce the sense of stalling during movement and ensure the safety of the mobile robot while traveling, the searched path needs to be smoothed to obtain a path that the mobile robot can track smoothly.

The path obtained after the pruning treatment is still connected by several path segments, and therefore does not form a smooth trajectory and fails to meet the safety requirements for smooth movement and no emergency stops of the vehicle. Therefore, the generated path still needs to be smoothed.

Among various types of spline curves, the B-spline curve is a widely used and highly flexible curve [26] also known as the Bezier curve. It was developed by Jacob Schoenberg as a mathematical method for describing curves. The turning points of a spline curve are controlled by local vertices within a certain range, so it is necessary to choose the appropriate control vertices. The functional formula of the B-spline curve is shown in Equation (4):

(4) $$P_{i,n}(u)=\sum_{i=1}^{n}P_{i+k}\cdot F_{k,n}(u)$$

where i denotes the serial number of the spline curve; i=1,2,3…n, n denotes the basis function parameter curve of the spline curve as the order n; $P_{i+k}$ denotes the k-th control point of the Bessel curve in the i paragraph; $F_{k,n}(u)$ is the basis function of the n-Bessel curve; u(0≤u≤1) represents the parameter of the Bessel curve, whose expression can be expressed as Equation (5):

(5) $$F_{k,n}(u)=C_{n}^{k}u^{k}{\left(1-u\right)}^{n-k},k=0,1,2,\ldots n$$

**Figure 5:**
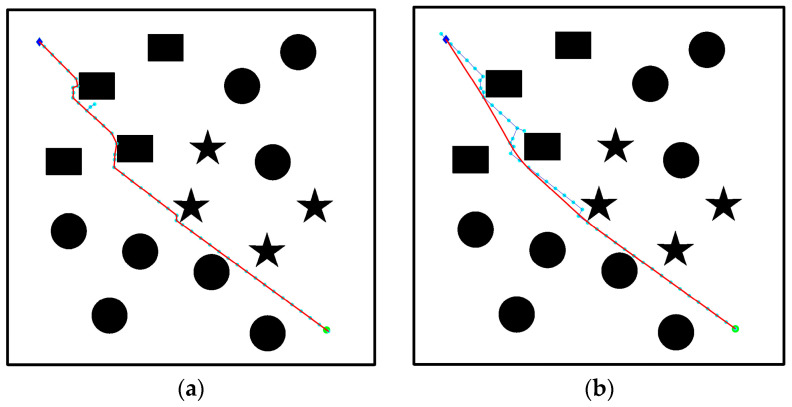
Path smoothing in different kinds of obstacle environments: (**a**) path smoothing is not added; (**b**) path smoothing is applied.

**Figure 6:**
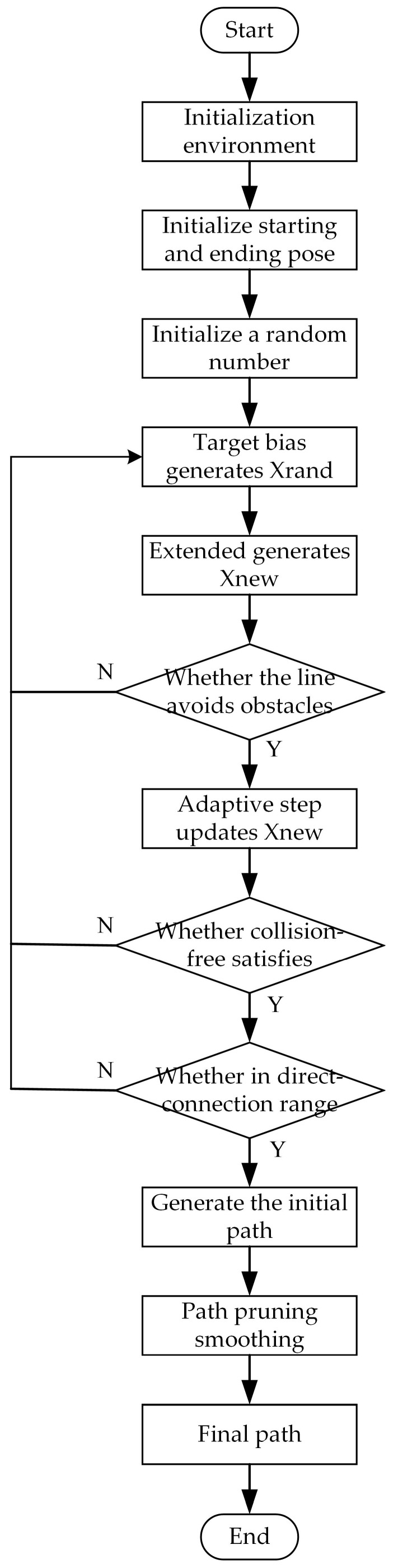
Improved RRT algorithm flow chart.

In Formula (6), the value n represents the smoothness of the Bessel curve, and the larger the value n, the higher the smoothness, but the complexity of calculation will also increase. Taking into account the complexity of the calculation and the smoothing requirements of the path, a cubic B-spline curve was selected in this paper for smoothing processing. The third-order Bessel curve could meet the pose and path smoothing requirements in this paper. The cubic B-spline curve basis function $F_{k,n}(u)$ of the car body substitutes n=3 into Equation (5) to obtain Equation (6). Figure 5 shows the path smoothing in different kinds of obstacle environments. A flow chart of the improved RRT algorithm considered in this paper is shown in Figure 6.

(6) $$\left\{ \begin{array}{l} F_{0,3}(u)=\frac{1}{6}(-u^{3}+3u^{2}-3u+1)\\ F_{1,3}(u)=\frac{1}{6}(3u^{3}-6u^{2}+4)\\ F_{2,3}(u)=\frac{1}{6}(-3u^{3}+3u^{2}+3u+1)\\ F_{3,3}(u)=\frac{1}{6}u^{3} \end{array}\right.$$

## 3. Comparative Analysis of Simulation Algorithm Experiment

In this paper, MATLAB was used to conduct simulation experiments on the improved RRT algorithm proposed herein. The test host was the Lenovo R9000P, which is equipped with an AMD R7-5800H processor running at a main frequency of 3.0 GHz and possesses a memory size of 32 GB.

**Figure 7:**
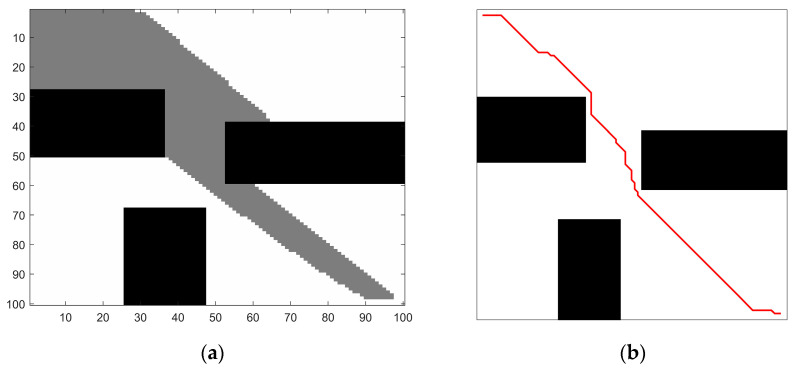
A* algorithm simulation: (**a**) pre-path planning; (**b**) post-path smoothing.

**Figure 8:**
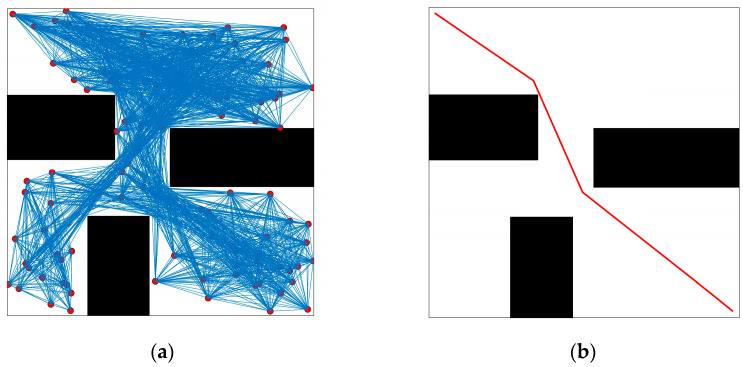
PRM algorithm simulation: (**a**) pre-path planning; (**b**) post-path smoothing.

**Figure 9:**
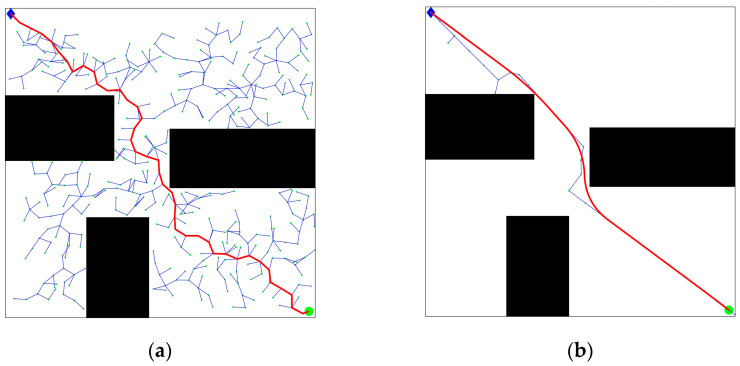
Simulation of RRT and improved RRT algorithm in the same environment: (**a**) RRT algorithm; (**b**) improved RRT algorithm.

Hart et al. invented the A* algorithm in 1968. This heuristic process combines the characteristics of Dijkstra’s algorithm and the best-first method. The A* algorithm uses heuristic data to guide the optimization process and offers excellent flexibility for various settings. As a result, it is widely used to identify efficient and effective systems in a short time. The algorithm evaluates the integrity of each node and is particularly useful for path planning in a known environment. On the other hand, the PRM (Probabilistic Road Map) algorithm is highly efficient for planning in high-dimensional spaces. The basic concept behind the PRM approach involves constructing a set of points, typically generated through spatial sampling. Figure 7, Figure 8 and Figure 9 compare the two algorithms with the RRT algorithm and its improved version. The comparison parameters for planning time, number of nodes and path length of the four algorithms in the obstacle environment are presented in Table 3. It can be observed from the experimental data that the improved RRT algorithm is significantly faster than both the A* and RRT algorithms in terms of time. It is 70.02% faster than the PRM algorithm and has 61% fewer sampling nodes. Additionally, the improved RRT algorithm shows a significant reduction in path length compared to the A*, PRM, and RRT algorithms. These results indicate that the improved RRT algorithm offers substantial advantages over traditional intelligent path planning algorithms. Next, simulations are carried out from the perspective of the RRT variant algorithm.

**Figure 10:**
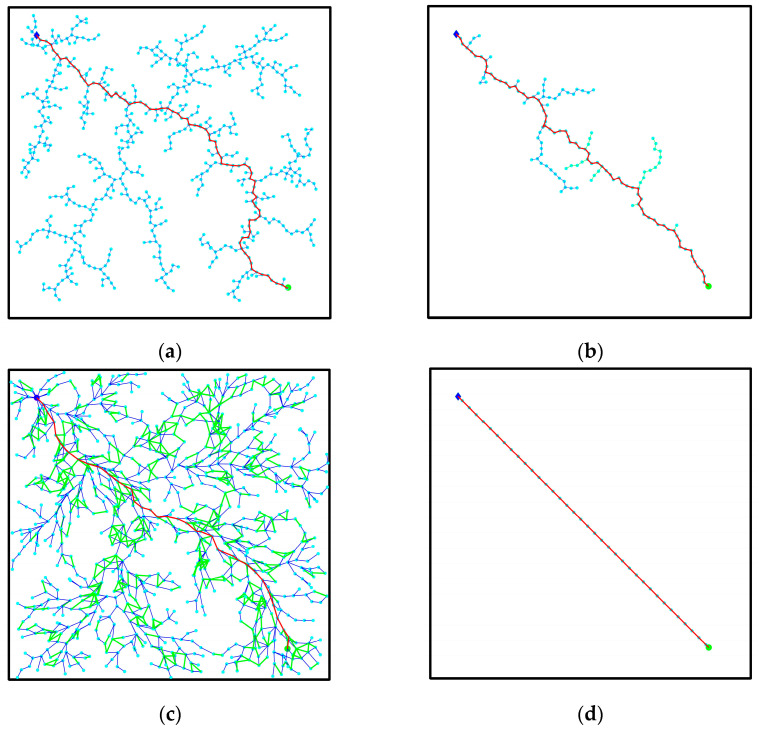
Comparison of algorithm effect in an obstruction-free environment: (**a**) RRT algorithm; (**b**) RRT-connect algorithm; (**c**) RRT* algorithm; (**d**) improved RRT algorithm.

**Figure 11:**
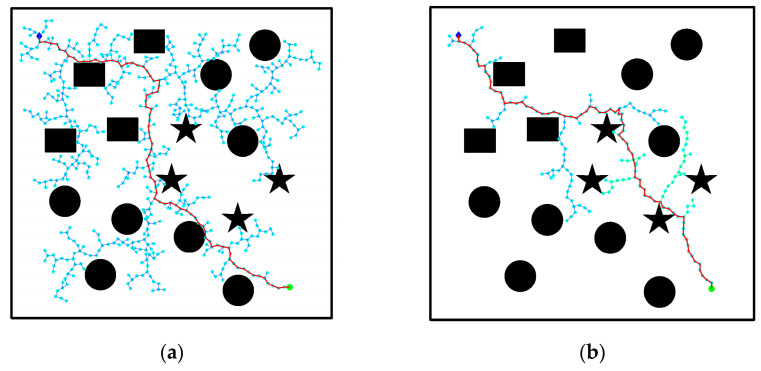

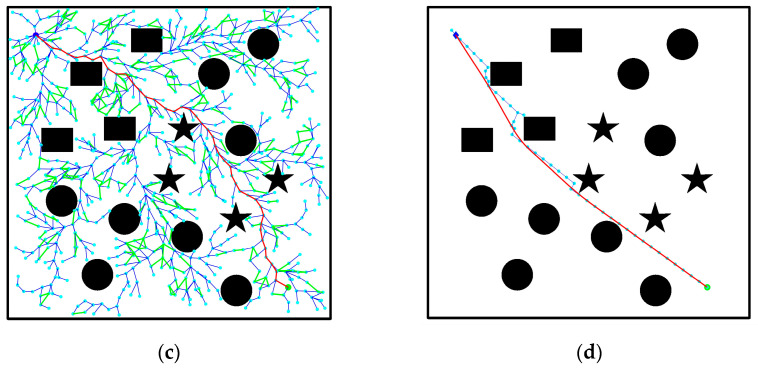
Comparison of algorithm effect in simple obstacle environment: (**a**) RRT algorithm; (**b**) RRT-connect algorithm; (**c**) RRT* algorithm; (**d**) improved RRT algorithm.

**Figure 12:**
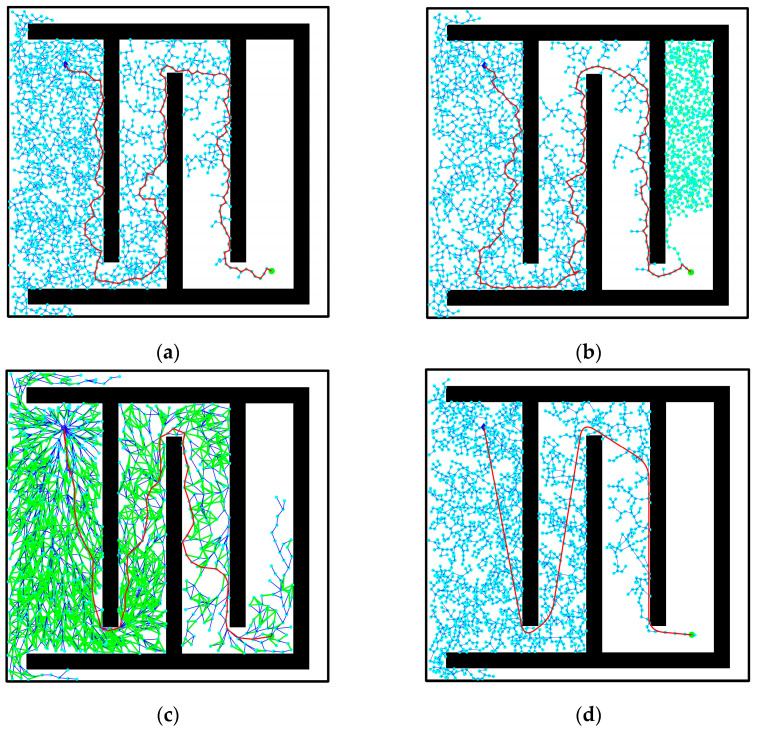
Comparison of algorithm effect in narrow channel environment: (**a**) RRT algorithm; (**b**) RRT-connect algorithm; (**c**) RRT* algorithm; (**d**) improved RRT algorithm.

**Figure 13:**
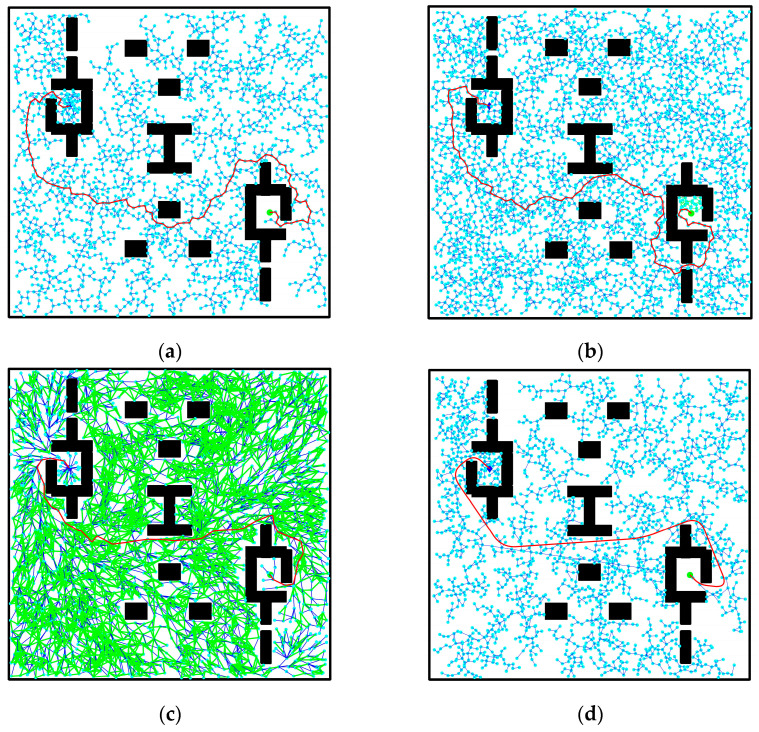
Comparison of algorithm effect in narrow entrance and exit environment: (**a**) RRT algorithm; (**b**) RRT-connect algorithm; (**c**) RRT* algorithm; (**d**) improved RRT algorithm.

To further verify the improved performance [27] of the RRT algorithm presented in this paper, a comparative test of global path planning was conducted using the RRT algorithm, RRT-connect algorithm [10], RRT* algorithm [12], and the improved RRT algorithm in four two-dimensional map environments: a barrier-free environment, a simple obstacle environment, a narrow channel obstacle environment, and a narrow starting point environment. The map size of each simulation environment was (1097, 1059), progressing from a general obstacle environment to the complex and narrow environments studied in this paper, thereby gradually validating the algorithm’s rationality. Fifty repeated experiments were performed for each method in the four environments, and the average value of each algorithm’s indices was calculated. The planning results of each algorithm in the four map environments are illustrated in Figure 10, Figure 11, Figure 12 and Figure 13: (a) depicts the planning effect of the traditional RRT algorithm, (b) displays the planning effect of the RRT-connect algorithm [28], (c) demonstrates the path planning effect of the RRT* algorithm, and (d) showcases the planning effect after path optimization using the improved RRT algorithm.

### 3.1. Obstruction-Free Environment

Figure 10 presents a comparison diagram of algorithm planning in an obstruction-free environment, with the starting point and target point set as (100,100) and (950,950), respectively. In Figure 10a, a feasible path planned by the basic RRT algorithm is depicted. Due to the blind search characteristic of the RRT algorithm, the growth tree is randomly distributed in the barrier-free area, resulting in low algorithm efficiency and poor path quality. Even in a barrier-free environment, a blind search is performed. Figure 10b shows a feasible path planned by the RRT-connect algorithm. The RRT-connect algorithm employs simultaneous sampling of the starting point and target points to significantly reduce the selection of sampling points. However, there are still many redundant points and issues with high path costs. Figure 10c illustrates a feasible path planned by the RRT* algorithm. The RRT* algorithm is an improvement upon the RRT algorithm, incorporating asymptotic optimization. Consequently, the RRT* algorithm achieves better path length and reduces part of the path cost. Nonetheless, it requires numerous repeated iterations, resulting in poor node utilization and search efficiency. It is characterized by blindness and relatively long iteration times. Figure 10d depicts a path realized by the improved RRT algorithm after path planning. The path eliminates redundant sampling points and accurately selects the optimal path in a barrier-free environment within a short timeframe. Its effect is significantly better than the previous path planning method.

The data in Table 4 clearly demonstrate that the map of the obstruction-free environment is simple, highlighting the significant advantages of the improved algorithm. The elimination of expanded sampling for useless nodes results in an accelerated search speed. In a barrier-free environment, the rapid search and expansion of the starting point and target point can be accurately achieved, allowing for optimal path selection. Comparing these results with those obtained using the RRT algorithm, RRT-connect algorithm, and RRT* algorithm, it can be observed that the number of extended nodes is reduced by 96.42%, 70.25%, and 96.40%, respectively. Additionally, the search time is reduced by 96.74%, 39.79%, and 84.81%, respectively. After node pruning and path smoothing optimization, the improved RRT algorithm exhibits a better path length. Specifically, it reduces the path length by 23.29% compared to the RRT algorithm, 17.67% compared to the RRT-connect algorithm, and 13.90% compared to the RRT* algorithm. Simulation results confirm that the improved algorithm outperforms other sampling algorithms.

### 3.2. Simple Obstacle Environment

Figure 11 displays the planning effect of the environment map with simple obstacles. The starting points and target points are set as (100,100) and (950, 950), respectively. Unlike the barrier-free environment depicted in Figure 10, obstacles are introduced here to evaluate the algorithm’s performance in an environment with basic obstacles. The environment contains obstacles of varying sizes, shapes, and relative positions, requiring different algorithms to perform static trajectory planning on the same environment map. In Figure 11a, the RRT algorithm shows the trajectory path. It can be observed that the expansion path of the random tree is relatively chaotic and tortuous, containing redundant nodes that the multi-node trajectory vehicle cannot accurately follow. Moving to Figure 11b, the RRT-connect algorithm samples the starting point and target point separately, resulting in a significant reduction in the selection of sampling points. However, there are still issues, such as the inability to follow a zigzag path and the presence of multiple redundant nodes. Figure 11c demonstrates a better planning effect compared to the RRT algorithm and RRT-connect algorithm. Although the iteration time for path planning is somewhat increased, the path cost is reduced, and the resulting path is relatively reasonable. Lastly, Figure 11d showcases the path effect planned by the improved RRT algorithm. The path appears smoother and exhibits noticeable optimization and improvement in terms of path cost length, number of nodes, and planning time.

The experimental results in a simple obstacle environment demonstrate that the improved RRT algorithm proposed in this paper exhibits a favorable path planning effect in such conditions. In comparison to the RRT algorithm, the planning time of the RRT-connect algorithm and RRT* algorithm is reduced by 91.44%, 10.10%, and 63.41%, respectively, demonstrating their significantly enhanced efficiency. Furthermore, the number of sampling nodes decreases by 89.62%, 83.10%, and 91.50%, respectively, leading to a substantial improvement in sampling efficiency. Moreover, the path length is reduced by 23.91%, 22.44%, and 18.65%, respectively, resulting in a decreased path cost and optimized initial path generation. Consequently, the improved RRT algorithm ensures relatively small path lengths in this environment, thus enhancing the algorithm’s planning efficiency. Additionally, the path demonstrates smoothness when navigating through environments with obstacles.

### 3.3. Narrow Channel Environment

Figure 12 illustrates the simulation effect in a narrow channel environment, with the starting point and target point positioned at (200,200) and (900,900), respectively. In contrast to the simple obstacle environment depicted in Figure 11, the path planning in Figure 12 takes place within a single narrow channel, without the inclusion of redundant obstacles. The primary objective is to observe whether the adaptive step size strategy will encounter local minima within the narrow channel environment and assess the optimization efficiency within a confined space. Upon comparing the planning effects of the four algorithms, it is evident that both the RRT algorithm and RRT-connect algorithm struggle to navigate small spaces, often falling into local dilemmas. These algorithms face challenges in swiftly searching for an exit, resulting in excessively long path tracing sampling times and an abundance of redundant paths. The RRT* algorithm, on the other hand, can adjust the path effect by appropriately increasing the number of iterations within narrow and complex environments or when multiple obstacles are present. As shown in Figure 11c, as 1000 iterations proved insufficient for the path planning requirements, an additional 4000 iterations are selected.

As a result, the path length is shorter than that of the RRT algorithm and RRT-connect algorithm. However, this approach leads to prolonged planning iteration times, and the path cannot be accurately tracked. In contrast, the improved RRT algorithm proposed in this paper utilizes a greedy algorithm to eliminate redundant nodes. It adopts target bias sampling to minimize the selection of sampling points, thus enhancing planning efficiency. Furthermore, the algorithm incorporates adaptive step size to improve path planning convergence speed, resulting in significant optimization effects. Consequently, the improved RRT algorithm swiftly generates a safe and collision-free feasible global path within the narrow channel environment.

According to the experimental data obtained in the narrow channel environment, the improved RRT algorithm demonstrates a planning time of 34.13 s, which is 76.05% shorter than that of the RRT algorithm, 55.09% shorter than that of the RRT-connect algorithm, and 59.24% shorter than that of the RRT* algorithm. It achieves a superior path and exhibits higher search efficiency. Furthermore, the number of nodes is 5880, which is 2.10% less than that of the RRT algorithm and 24.34% less than that of the RRT-connect algorithm, effectively eliminating redundant sampling points. Concerning path length, it is reduced by 23.61% compared to the RRT algorithm, 19.66% compared to the RRT-connect algorithm, and 6.68% compared to the RRT* algorithm. As depicted in Figure 12, the path appears smooth while maintaining a small path cost, demonstrating the effective optimization in path planning achieved by the algorithm.

### 3.4. Narrow Entrance and Exit Environment

Figure 13 presents the simulation effect in a complex narrow entrance and exit obstacle environment. The starting point and target point are set as (210,340) and (890,700), respectively. In contrast to the previous three cases, the environment in Figure 13 primarily emphasizes the narrowness near the starting and ending pose points, resulting in smaller entrances and exits. This increases the difficulty of path sampling and searching. The environment map simulates the narrow starting and ending positions typically encountered in reality, such as narrow elevator environments within corridors. This verifies the improved algorithm’s realism, generality, and applicability. Within the narrow channel environment, the RRT algorithm requires considerable time to select path sampling points, resulting in a large number of redundant sampling points. The generated path appears tortuous, and the associated path cost is unreasonably high. In comparison, the RRT-connect algorithm reduces the number of sampling points and shortens path planning time. It navigates the narrow environment relatively smoothly, but the quality of the planned path remains subpar. As for the RRT* algorithm, it exhibits a low probability of generating a path trajectory after 1000 iterations of sampling, as depicted in Figure 13. Consequently, the number of iteration samplings is increased to 4000 times. This leads to increased planning iteration time, reduced rapidity, decreased path cost, and improved path quality. Upon comparison, it becomes evident that the improved RRT algorithm presented in this paper can swiftly search for a smooth path with a reasonable path selection and lower path cost compared to other algorithms. It effectively navigates obstacle environments within a short duration and accomplishes optimal global path selection through narrow channels.

**Figure 14:**
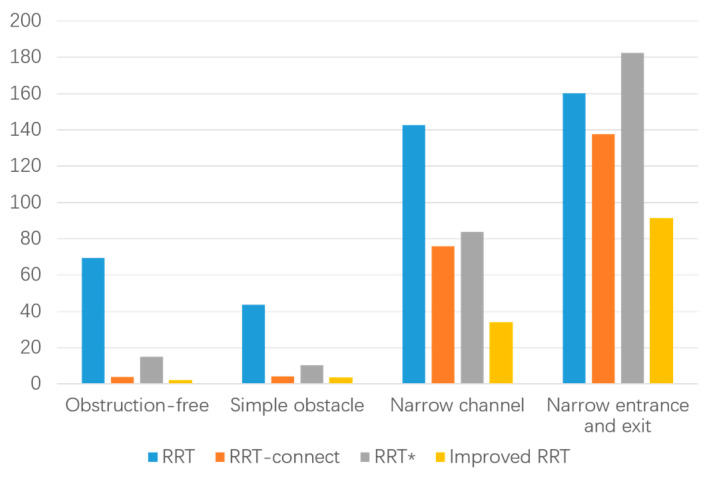
Comparison of processing time in different environments.

**Figure 15:**
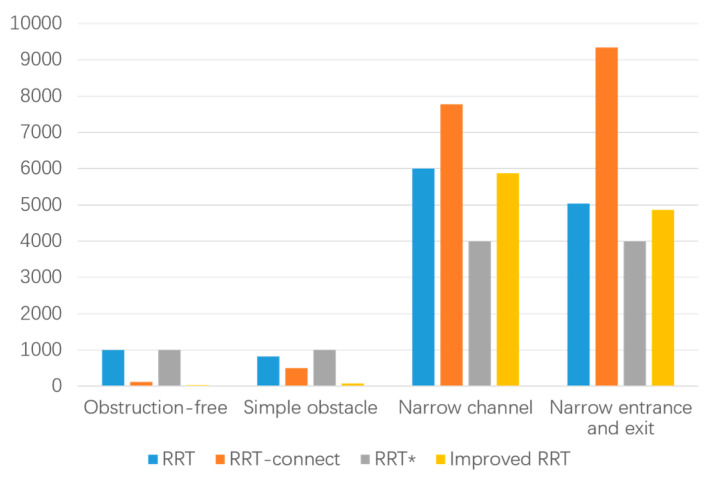
Comparison of sampling nodes in different environments.

**Figure 16:**
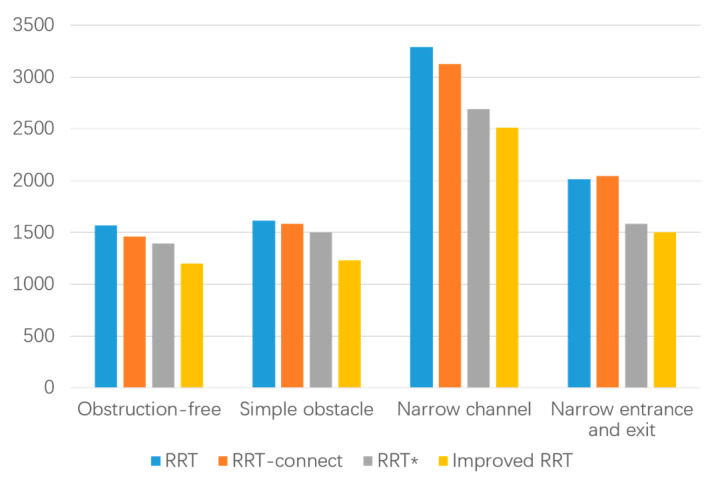
Comparison of path lengths in different environments.

In the data table of experimental results in the narrow environment, it is observed that the number of extended nodes in the improved RRT algorithm is slightly higher than that of the RRT* algorithm when traversing the entire environment map. However, it is reduced by 3.53% compared to the RRT algorithm and 47.97% to the RRT-connect algorithm, while also decreasing the number of sampling points. This allows for a more extensive search effect in a larger space. Regarding planning time, the improved algorithm demonstrates a 42.98% reduction compared to the RRT algorithm, a 33.53% reduction compared to the RRT-connect algorithm, and a 49.88% reduction compared to the RRT* algorithm. These results affirm that the improved algorithm addresses the speed deficiency of the RRT algorithm and meets the demands for increased speed. Furthermore, the path length achieved by the improved algorithm is strictly shorter than that of both the RRT and RRT-connect algorithms and slightly shorter than that of the RRT* algorithm. Additionally, in the narrow starting and ending point environment depicted in Figure 13, a smooth, trackable, and collision-free path is successfully planned. The path simulation results are displayed in Figure 13d. Detailed experimental data for the four environments can be found in Figure 14, Figure 15 and Figure 16 and Table 4.

It can be observed from the experimental path planning diagram and simulation data that the improved RRT algorithm presented in this paper exhibits clear advantages over the RRT algorithm, RRT-connect algorithm, and RRT* algorithm in terms of path planning time, the number of extended sampling nodes, and path cost. By employing the improved RRT algorithm for pruning and smoothing the path, the resulting trajectory remains smooth and unaffected by the presence of obstacles. The algorithm ensures that there are no collisions or restrictions at corners, thereby guaranteeing the safety and stability of the mobile robot during movement. Consequently, the proposed improved RRT algorithm in this paper offers higher path exploration efficiency, shorter path length, and a smooth and secure trajectory in four distinct obstacle environments.

## 4. Improved Algorithm Physical Verification

**Figure 17:**
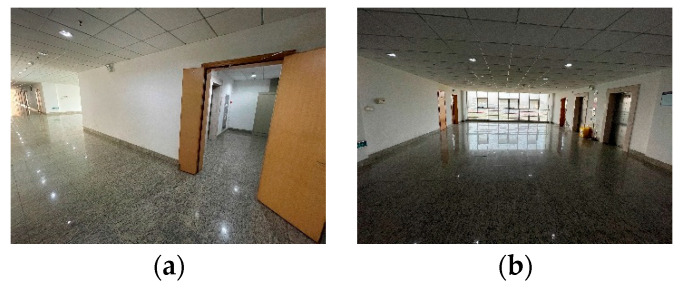
Experimental environment: (**a**) starting and ending point environment; (**b**) corridor environment.

The experimental test [29] environment was the sixth floor of the Yifu Science and Technology Building at China Jiliang University. The environmental map, which is shown in the form of portable gray map depicted in Figure 17, illustrates the elevator entrance at the starting point, the corridor along the path, and the elevator entrance at the target point, respectively. The corridor measures approximately 55 m in length and 3 m in width. For the test scenario, two elevator ports were strategically placed to simulate narrow environments at both the starting and target points.

**Figure 18:**
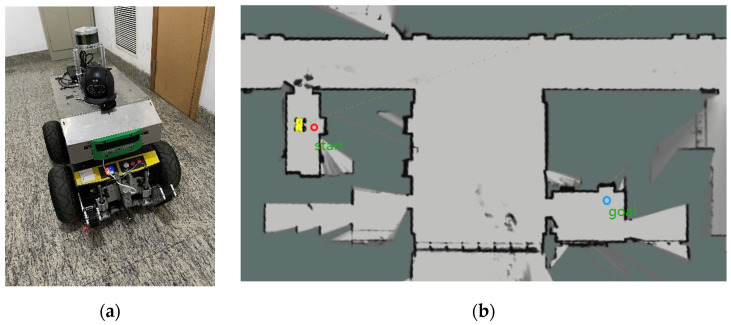
Experimental conditions: (**a**) mobile robot body; (**b**) corridor environment simulation.

The physical verification of the mobile robot’s path planning was conducted using the visualization tool Rviz, which operates under the Robot Operating System (ROS). The body structure of the insect-like mobile robot is depicted in Figure 18a. In the Rviz visual tool interface, the body is equipped with an external sensor called Lidar, as well as an internal sensor called IMU and an odometer. By utilizing pose collection point cloud data and SLAM technology, the Cartographer algorithm was employed to construct two-dimensional environment maps to simulate the experimental conditions of the vehicle’s surroundings. Figure 18b illustrates this, where the red circle represents the starting point and the blue circle represents the target point. These positions correspond to the two elevator exits within the narrow space of the actual corridor entrance. The boundary of the obstacle is depicted by the black line, while the white area represents the robot’s free movement space.

**Figure 19:**
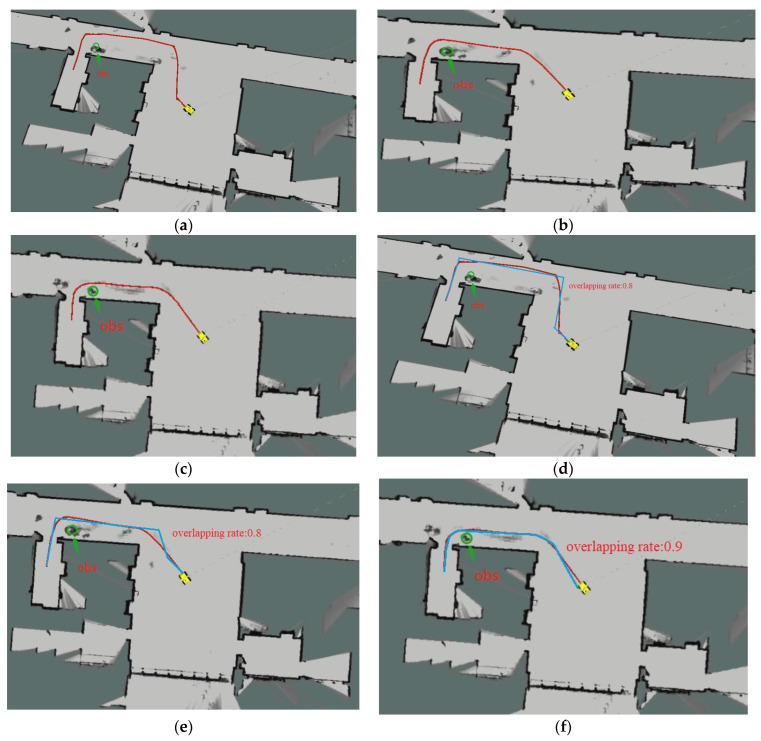
Comparison of algorithms in narrow corridor environment: (**a**) RRT algorithm; (**b**) RRT-connect algorithm; (**c**) improved RRT algorithm; (**d**) actual path of RRT algorithm; (**e**) actual path of RRT-connect algorithm; (**f**) actual path of improved RRT algorithm.

The three figures in Figure 19a–c, respectively, show the physical verification simulation diagrams of the RRT algorithm, RRT-connect algorithm, and the improved RRT algorithm after encountering static obstacles in the narrow channel environment. Figure 19d–f show the actual path in blue and the overlapping rate of both paths. The green circle in the figures represents the position of the obstacle set. From the experimental data and algorithm simulation diagrams, we can see that the improved algorithm’s path is more suitable for mobile robot tracking, with a shorter path length and less planning time. This confirms the superiority of the improved algorithm presented in this paper compared to other variants of RRT algorithms.

**Figure 20:**
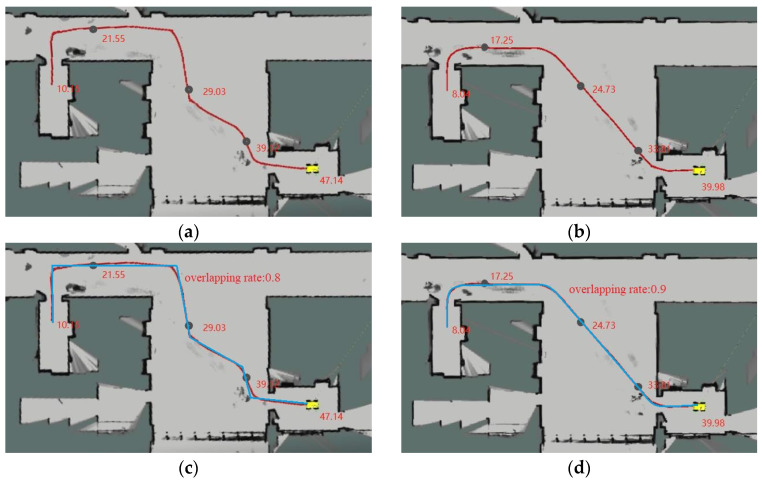
Physical verification: (**a**) RRT algorithm physical simulation; (**b**) improved RRT algorithm physical simulation; (**c**) actual path of RRT algorithm; (**d**) actual path of Improved RRT algorithm.

Figure 20a depicts the trajectory of the RRT algorithm [30] in location planning for two elevator ports. Although overall path planning is achieved, the randomness of the RRT algorithm prolongs the time required for path exploration [31]. Additionally, the excessive node sampling results in a suboptimal fit of the planned path to the obstacles, leading to longer path lengths and higher costs. However, the improved algorithm shows significant enhancements in these aspects. The initial path generated by the RRT algorithm exhibits a delayed response, causing noticeable frustration during mechanical obstacle avoidance and lacking a smoothing effect, which is unsuitable for the robot’s movement tracking. In contrast, the improved algorithm offers a smoother, safer, and more stable path, making it more suitable for vehicle body control. Figure 20b demonstrates the effectiveness of the proposed algorithm as a global path planning algorithm and Table 5 shows the results of environmental planning data. Figure 20c,d show the actual path in blue and the overlapping rate of both paths. Applying the improved RRT algorithm to the robot results in significant reductions in planning time and accelerated convergence speed. The algorithm employs the target offset sampling method to minimize the generation of redundant sampling points, thereby reducing sampling time and improving the accuracy of point selection. The resulting moving path is better suited for navigating around obstacles. By addressing the limitation of a fixed expansion step size in traditional RRT algorithms, the adaptive step size of the improved algorithm dynamically selects sampling points based on the actual situation and adjusts the sampling distance. This approach enhances the speed of path search, reduces the randomness in path generation, and improves the stability and efficiency of the algorithm. The improved algorithm yields smoother and higher-quality paths, making it more suitable for the robot to follow.

## 5. Conclusions

In this paper, we propose an improved RRT algorithm based on adaptive step size adjustment to address the challenges faced by the traditional RRT algorithm in global path planning for mobile robots. These challenges include an excessive number of redundant sampling nodes, slow convergence, low search efficiency, and uneven path transitions. In most cases, the optimal selection of sampling nodes involves setting thresholds or assigning weights and it is limited by environmental conditions and usage scenarios, and most of the steps will use the idea of cumulative iteration because of the convenience of superposition. This paper begins by considering the basic formula of generating new sampling nodes, and the threshold value is not involved. Then, it focuses on the obstacle environment, avoids falling into the local minimum situation in the narrow environment, and improves the optimal quality of sampling points, which also improves the rationality of path step size selection. Firstly, the algorithm initializes the environment and determines the starting and ending pose information. It introduces the target bias sampling method to generate random sampling points closer to the target point and expands the exploration towards the target direction to generate new nodes. Secondly, to overcome the limitation of the traditional RRT algorithm’s fixed step size for global traversal, we adopt an adaptive step size strategy to adjust the length of tree extensions according to the obstacle environment. Compared to the RRT algorithm, RRT-connect algorithm, and RRT* algorithm, our approach reduces the planning time by 42.98%, 33.53%, and 49.88%, respectively. The generated path is better suited to the obstacle environment and has a lower path cost. Additionally, angle and collision constraints are detected during robot movement, and the initial path is extended accordingly. Finally, the initial path is smoothed using pruning and B-spline sampling techniques to simplify it and eliminate redundant nodes. The resulting planned path is smoother and safer. We validate the algorithm by implementing it on a mobile robot in a corridor environment, which confirms its feasibility. Through a comparison of experimental data, we observe significant improvements in search time, sampling cost, and path cost for the paths generated by our improved RRT algorithm in four different environments. In daily life, this improved algorithm can accomplish path planning more efficiently, especially in scenarios involving complex obstacles, narrow channels, and limited starting and ending positions. It preserves the advantages of the original algorithm and serves as a foundation for local obstacle avoidance algorithms and dynamic obstacle fusion in the environment. Its practical significance for real-life global path planning of mobile robots is evident. While the algorithm presented in this paper has enhanced the path planning, it focuses on static obstacles; thus, its effectiveness in avoiding dynamic obstacles remains unknown. Additionally, the simulations and experiments conducted in this study were limited to a two-dimensional environment. It is well known that the RRT algorithm effectively operates in three-dimensional environments. Therefore, the improvement in path planning effectiveness within a 3D space shown here is not remarkable.

## Figures and Tables

**Table 1:** Comparison of environmental map models.

Environmental Map	Advantages	Shortcomings
grid map	Grid map environment information is more accurate, structured, and ordered, and it is convenient for constructing models.	A lot of memory usage; map obstacle environment too regular.
feature map	Uses feature geometry to represent maps; geometric shapes are described as infinitely fine with no extra space taken up.	Cannot represent spatial distribution; a highly structured environment is more suitable.
topological map	Composed of nodes and edges; can be accompanied by weights; more suitable for immediate expansion; suitable for expressing the direct relationship between each position.	Lack of feature information; mostly used for path planning; not often used for map construction.

**Table 2:** Comparison before and after adding adaptive step size.

Index	RRT	Improved RRT
number of nodes	671	56
time (s)	37.43	2.52

**Table 3:** Simulation parameters of four algorithms.

Environment	Index	A*	PRM	RRT	Improved RRT
Obstacle environment	time (s)	46.60	5.57	26.76	1.67
	number of nodes	/	100	576	39
	length (cm)	731.54	707.27	810.32	699.82

**Table 4:** Simulation parameters of four environmental experiments.

Environment	Index	RRT	RRT-Connect	RRT*	Improved RRT
Obstruction-free environment	time (s)	69.40	3.76	14.88	2.26
	number of nodes	1005	121	1000	36
	length (cm)	1567.1	1460.1	1396.1	1202.1
Simple obstacle environment	time (s)	43.69	4.16	10.22	3.74
	number of nodes	819	503	1000	85
	length (cm)	1615.9	1585.3	1500.7	1229.6
Narrow channel environment	time (s)	142.49	76.00	83.74	34.13
	number of nodes	6006	7772	4000	5880
	length (cm)	3289.0	3127.3	2692.3	2512.5
Narrow entrance and exit environment	time (s)	160.33	137.53	182.39	91.42
	number of nodes	5040	9345	4000	4862
	length (cm)	2015.0	2045.1	1583.8	1504.8

**Table 5:** Comparison of path planning data.

Index	RRT	RRT-Connect	Improved RRT
length (m)	58.78	56.12	51.70
time (s)	47.14	43.25	39.98

## Data Availability

The data used to support the findings of the study, which is not applicable for studies not involving humans or animals, can be downloaded from https://pan.baidu.com/s/1SOQKOooktsKWFR-QNla_Zg?pwd=bvdc, accessed on 1 June 2023, and the deadline is June 29.
